# The Paradoxes of Viral mRNA Translation during Mammalian Orthoreovirus Infection

**DOI:** 10.3390/v13020275

**Published:** 2021-02-11

**Authors:** Yingying Guo, John S. L. Parker

**Affiliations:** Baker Institute for Animal Health, College of Veterinary Medicine, Cornell University, Ithaca, New York, NY 14853, USA; yg264@cornell.edu

**Keywords:** reovirus, translation, integrated stress response, stress granules, virus factories, PKR, RNase L

## Abstract

De novo viral protein synthesis following entry into host cells is essential for viral replication. As a consequence, viruses have evolved mechanisms to engage the host translational machinery while at the same time avoiding or counteracting host defenses that act to repress translation. Mammalian orthoreoviruses are dsRNA-containing viruses whose mRNAs were used as models for early investigations into the mechanisms that underpin the recognition and engagement of eukaryotic mRNAs by host cell ribosomes. However, there remain many unanswered questions and paradoxes regarding translation of reoviral mRNAs in the context of infection. This review summarizes the current state of knowledge about reovirus translation, identifies key unanswered questions, and proposes possible pathways toward a better understanding of reovirus translation.

## 1. Introduction

Viruses must use the host translation machinery to synthesize viral proteins. Early in infection viral mRNA transcripts must compete with a large excess of host transcripts for access to the translation machinery. Defective viral translation will severely impact viral replication. Eukaryotic translation is a complex and sophisticated cellular process which includes four phases: initiation, elongation, termination, and recycling [1,2]. The translation of mRNA into new proteins consumes considerable energy, and cellular translation is tightly controlled during cell proliferation and growth, especially when cells are stressed [1,3]. As a consequence, host antiviral responses are integrated with suppression of host translation to constrain viral replication [4,5,6,7]. Viruses have evolved a variety of mechanisms to overcome the translational constraints induced by the host antiviral response.

Mammalian orthoreoviruses (reoviruses) are nonenveloped and carry a segmented double-stranded (ds)RNA genome within an icosahedral core particle. A second outer-capsid layer that functions to mediate virus entry surrounds the core [8]. Core particles are deposited in the cytoplasm following virus entry and synthesize viral mRNAs which are released into the cytoplasm. The viral mRNAs are capped but are not polyadenylated [9,10]. Ten species of mRNA are synthesized from the ten dsRNA genome segments. The 10 viral mRNAs encode 12 viral proteins (Table 1). Most viral mRNAs encode a single viral protein. However, the *s1* mRNA encodes two proteins: the attachment protein, σ1, and a second nonstructural protein called σ1s, which is synthesized from an alternative reading frame by “leaky scanning” and initiation on a downstream methionine [11,12,13,14,15]. The *m3* mRNA encodes the viral nonstructural protein μNS. It is believed that an N-terminally deleted form of μNS called μNSC is synthesized by alternative initiation at a downstream methionine. However, this has not been experimentally confirmed for mammalian orthoreoviruses.

This review focuses on what is currently known about translation of reovirus mRNA. Our goals are to identify outstanding questions and to highlight the implications of recent findings regarding the compartmentalization of translation in reovirus-infected cells.

## 2. Reovirus mRNA Structure and Stability

Reovirus mRNAs are synthesized by viral RNA-dependent RNA polymerases (λ3 protein) within viral core particles using the negative-sense strand of each dsRNA gene segment as a template. The viral mRNAs are capped at their 5′ ends by guanylyl- and methyl-transferase enzymes to form a eukaryotic cap 1 structure as they are extruded into the cytoplasm [16,17,18]. The capping enzyme activities reside within the λ2 structural protein, five copies of which form turret-like structures at each of the five-fold axes of symmetry of the core [19]. Although they are capped, all reoviral mRNAs lack a poly(A) tail. Short conserved sequences are present at the 5′ (GCAT) and 3′ (UCAUC) ends of each viral mRNA.

Polyadenylation of the 3′ end stabilizes mRNAs and protects them from degradation by the cellular mRNA decay machinery, which targets mRNAs without a 3′-poly(A) tail for decapping and degradation by 5′ and 3′ exonucleases [20]. Lacking a 3′-poly(A) tail, reovirus mRNAs should be targets for the cellular mRNA decay machinery. However, the stability of reovirus mRNAs within cells has not been examined in detail. Several positive-strand RNA virus genomes lack a 3′ poly(A), but avoid degradation by having cis-acting stem-loop structures at their 3′ ends that inhibit the normal mRNA decay pathways [21]. Early after reovirus infection, transcripts from only 4 of the 10 gene segments (L1, M3, S3, and S4) can be detected, whereas at later times all 10 mRNA transcripts are detected [22]. These findings led to a hypothesis that early transcription differs from late transcription and that the transcription of some genes may be suppressed by a cellular factor. However, in vitro core transcription assays show that all gene segments are equally transcribed [23], suggesting either that transcriptionally active core particles prepared in vitro differ from those that are deposited into the cytosol of infected cells or that some other explanation for the difference in transcript levels early in infection is at play. One possibility is that viral mRNAs differ in their stability in the absence of other viral proteins; this hypothesis has not as yet been carefully evaluated. If true, then it is possible that one or more viral proteins synthesized from the four “early transcripts” stabilize the remaining viral transcripts. In support of this hypothesis, recent findings have shown that the viral single-stranded RNA-binding protein σNS, encoded by the *s3* mRNA, stabilizes viral RNAs [24]. σNS interacts with μNS, a nonstructural protein also synthesized from one of the “early transcripts” and is responsible for forming viral factories (VFs). VFs are compartments within reovirus infected cells that are the sites of viral replication and assembly. Viral RNAs localize and concentrate within VFs and it is possible that sequestration of viral mRNA within these structures may also protect them from degradation. Further evidence supporting this idea is that reovirus core particles are rapidly embedded within VFs formed by μNS [25,26].

Canonical eukaryotic mRNAs are circularized during translation by the poly(A)-binding protein (PABP) which binds to both the 3′ poly(A) and the eIF4F complex. Circularization of mRNA during translation enhances translation [27]. For rotaviruses, another member of the Reoviridae family, the NSP3 protein substitutes for PABP by specifically binding the four-nucleotide conserved 3′ sequence at the end of rotaviral mRNAs and also interacting with eIF4F [28,29]. In contrast, reoviruses have no viral protein known to specifically interact with the conserved 3′ sequence found at the end of reovirus mRNAs. Reoviruses express a single-stranded RNA-binding protein, σNS, that binds nonspecifically to viral RNA [24,30,31]. It is possible that circularization is not required for efficient reoviral mRNA translation or that circularization is mediated in some other way, perhaps by interaction with a yet-unidentified cellular factor. However, there are no conserved RNA structures at the 3′ end of reovirus mRNAs that might mediate an interaction with a putative cellular factor.

Studies in the 1970s of the structure of reovirus mRNA 5′ termini and their binding by ribosomes contributed to our current understanding of eukaryotic cap-dependent translation and ribosomal scanning [32,33,34,35,36,37]. Ribosome protection assays using wheat germ extracts and reovirus mRNAs were key to uncovering the important roles of the 5′ terminal cap in stabilizing mRNA and enhancing ribosome attachment to initiate translation.

The 5′ and 3′ untranslated regions (UTRs) of reovirus mRNAs are short. The 5′ UTRs vary between 12 and 32 nucleotides in length, and the 3′ UTRs vary between 35 and 83 nt in length (Table 1). A ribosomal “footprint” is between 28–32 nt, with the P site for the start codon lying approximately in the middle of this footprint (14–15 nucleotides from the trailing edge) [38]. The very short length of the 5′ UTRs of some reovirus mRNAs raises a question of how translation of reovirus mRNAs is initiated. For example, the *s1* reovirus mRNA has a 5′ UTR that is 12 nucleotides long. In order to place the initiating methionine tRNA at the P-site would require that the footprint of the ribosome overhang the cap structure. If the canonical eIF4F initiation complex were associated with the cap, this would also provide a steric hindrance to proper initiation at the start codon and joining of the 60S large ribosomal subunit. Further work to identify the specific initiation factors associated with reovirus mRNAs, particularly those with short 5′ UTRs, may help to uncover the mechanisms behind translational initiation.

## 3. Challenges for Viral Translation Early in Infection

Newly synthesized reovirus mRNAs are deposited into the cytoplasm absent any of the RNA-binding proteins that normally associate with cellular mRNAs [39]. Naked mRNAs are not found within normal cells, are easily targeted for degradation, and can adopt folds that contain stretches of dsRNA that can activate cellular pattern-recognition receptors [40]. Indeed, in vitro purified s1 reovirus mRNA is a potent activator of dsRNA-activated protein kinase R (PKR) [41]. The capped mRNAs extruded into the cytoplasm from core particles must engage 43S preinitiation complexes to initiate translation. De novo synthesis of viral proteins is required to establish viral replication sites or factories that incorporate the transcribing core particles. Diffusion of viral mRNAs and proteins away from core particles presents a challenge to organizing a replication site centered around the incoming viral cores.

Stress granules (SGs) are dynamic structures that contain messenger ribonucleoprotein (mRNP) complexes and a variety of other proteins including RNA-binding proteins. SGs are liquid–liquid phase-separation structures that form rapidly in response to stress-activated signal-pathway-mediated stalling of translational initiation and that dissipate upon removal of the stress [42]. Virus infection can induce translational stress by the activation of eIF2 kinases or by the cleavage or inactivation of translation-initiation factors leading to inhibition of translation initiation [39,43]. Stalled translation initiation leads to the disassembly of polysomes and the release of 48S ribosome mRNP complexes. It is thought that naked regions of mRNAs exposed upon dissociation of polysomes leads to RNA–RNA interactions that are important drivers of SG assembly [44,45]. Subsequent crosslinking of RNAs by multivalent RNA-binding proteins and their self-oligomerization and condensation leads to the liquid–liquid-phase separation that drives SG formation [46]. Among the various RNA binding proteins found in SGs, G3BP1 plays a central role as a molecular switch that triggers the initial phase separation that leads to SG assembly [47,48]. The carboxy terminus of G3BP1 interacts with free RNAs and 40S ribosomal subunits [49]. Three intrinsically disordered regions (IDRs) within G3BP1 regulate and tune the liquid–liquid phase separation. Phosphorylation within the IDRs regulates the propensity of G3BP1 to undergo phase separation and thus regulate SG dynamics [47].

SGs sequester stalled ribosomal complexes that lacked a ternary complex. SGs can stabilize certain mRNAs that collect within them until the stress can be resolved or target others for degradation within processing bodies [50,51]. In addition, SGs may serve as a signaling platform for the initiation of antiviral responses. Multiple signaling molecules involved in antiviral responses localize to SGs, including PKR, RNase L, RIG-I, MDA-5, and others. In general, because of the translational repression accompanying SG formation and the dependence of viruses on host translation machinery, most viruses appear to antagonize SG formation. However, there are exceptions with some viruses tolerating the presence of SGs or exploiting SG responses to benefit viral replication [52,53,54,55].

Reovirus infection induces SG formation as early as 2 h after infection, with peak SG numbers seen approximately 6 h after infection. However, as the infection progresses SGs disappear, and the infected cells become refractory to further induction of SGs [25,54]. The initial formation of SGs correlates with and requires phosphorylation of eukaryotic translation initiation factor 2 subunit α (eIF2α) as SGs do not form in mouse embryonic fibroblasts expressing a mutant form of eIF2α that cannot be phosphorylated. These observations suggest that early after infection, stress kinases responsible for eIF2α phosphorylation are activated. Removal of the viral outer-capsid proteins during virus entry is required for the induction of SG formation, but viral transcription and translation are not required [25]. Interestingly, experiments to identify which of the four known eIF2α kinases are involved found that mouse embryonic fibroblasts individually lacking PKR, PERK, HRI, or GCN2 still formed SGs early after reovirus infection, suggesting that more than one of these kinases is activated early after virus entry [25]. Which kinases in addition to PKR are activated and what viral factor or factors are responsible for their activation is an important outstanding question.

Viral core particles localize to SGs soon after they enter the cytoplasm [25]. Unlike the induction of SG formation, localization of viral core particles to SGs does require active viral transcription [25]. This observation infers that viral mRNAs are responsible for the recruitment of viral cores to SGs. It is possible that viral mRNA–mRNA interactions drive the formation of small nascent SGs that surround the transcribing core particles. Subsequently, these smaller core-containing SGs may be actively recruited to larger SGs in a microtubule-dependent manner [56]. Further experimentation in this area should resolve these questions. 

Intriguingly, inducing SG formation in cells by treatment with sodium arsenite 30 min prior to infection benefits subsequent reovirus replication [54]. Sodium arsenite activates the heme-regulated inhibitor kinase (HRI) which phosphorylates eIF2α [57]. Cells pretreated with sodium arsenite showed higher levels of viral protein synthesis and higher percentages of infected cells and viral titer in a cell-type-specific manner [54]. Given that viral core particles localize to SGs, these observations suggest that translation of reoviral mRNAs is not suppressed by SG formation at early times after infection. SGs contain a higher local concentration of translation-initiation factors and other factors required for translation. Although, it has long been assumed that active translation does not occur within SGs, recent findings indicate that cellular mRNAs can be actively translated within SGs, indicating that the environment within SGs is not itself inhibitory to translation [58]. SGs can also protect mRNA from degradation and serve as a reservoir of translational machinery that may be usurped by the virus for translation of its own mRNAs. One advantage to this would be that viral transcripts and proteins are concentrated and sequestered at very early times postinfection and prior to the formation of VFs. SGs could provide a protected environment that allows initial viral mRNA translation and prevents the diffusion of viral proteins and mRNAs away from the core particles. It may be that SGs act as niduses for the subsequent formation of VFs. Supporting this idea is the observation that the viral nonstructural protein μNS, which forms the matrix of VFs, colocalizes with SG markers at early times after infection (6 h postinfection). Amino acid residues 78 and 79 in μNS are responsible for the localization of μNS with SGs. These residues overlap with a region of μNS responsible for interaction with the core structural protein, λ2. μNS is itself unable to suppress SG formation [59].

## 4. Reovirus and the Integrated Stress Response (ISR)

The ISR is a cellular signaling pathway that activates transcription of a set of genes responsible for ameliorating and adapting to the initiating stress. The ISR signaling begins with phosphorylation of eIF2α and inhibition of translational initiation. Counterintuitively, a subset of mRNAs are translated preferentially when eIF2α is phosphorylated. This subset includes key transcription factors (e.g., ATF4, CHOP, etc.) that act to up-regulate genes that function to allow cells to adapt to and ameliorate the underlying stress. The mRNAs of ISR genes often have short upstream open reading frames (uORFs) in their 5′ untranslated regions that act to inhibit translation under normal concentrations of ternary complex. However, when translation initiation is inhibited and the concentrations of ternary complex become limiting, ribosomes scan past the uORFs and initiate at the canonical start codon of these mRNAs leading to synthesis of ISR proteins [60]. 

Reovirus infection activates and appears to benefit from phosphorylation of eIF2α and activation of the ISR [61]. Unlike most other viruses, reoviruses replicate less efficiently in mouse embryonic fibroblast cells that lack eIF2α kinases or that express a non-phosphorylatable mutant of eIF2α (eIF2αS51A) [54,61]. As mentioned earlier, it is possible that reovirus infection benefits from SG formation early after virus entry. However, the exact mechanism by which reoviruses benefit from activation of the ISR remains unknown. 

Later in reovirus infection, SGs disassemble, despite the presence of high levels of phosphorylated eIF2α [62]. Viral gene expression is required for SG disassembly, indicating that one or more viral proteins are involved [62]. As mentioned previously, the reovirus nonstructural protein μNS colocalizes with SGs at early times postinfection. Ectopically expressed μNS also colocalizes with sodium arsenite-induced SGs [59]. However, ectopic expression of μNS is not sufficient to disassemble preformed SGs [59]. The SG-associated proteins G3BP1, Caprin1, USP10, TIAR, TIA-1, and eIF3b localize to the periphery of VFs in a subset of infected cells. The localization of these SG proteins is dependent upon an interaction between σNS and the C-terminus of G3BP1 and is also dependent upon polysome dissociation [63]. The interaction between G3BP1 and σNS is important for the relocalization of SG proteins from canonical SGs to the periphery of virus factories and may explain the disruption of SGs that occurs later in infection. Indeed, overexpression of σNS and μNS together disrupts the capacity of sodium arsenite to induce SGs. The findings that SG components relocalize to the margins of VFs, together with the benefits that reovirus replication accrues from activation of the ISR, further support the idea that SGs serve as a reservoir of translational machinery that is used by the virus within VFs for its own translation. Alphaviruses appear to exploit SGs in similar ways [55]. Chikungunya virus nonstructural protein nsP3 recruits G3BP1 into cytoplasmic foci that do not contain other canonical SG proteins [64]. The recruitment of G3BP1 and its homolog G3BP2 by nsP3 into cytoplasmic foci benefits Chikungunya virus replication and nsP3 uses G3BP proteins as a means to concentrate the viral replication complexes and to recruit the translation-initiation machinery [65,66]. Our recent findings indicate that the reovirus dsRNA-binding protein σ3 may also be involved in preventing SG formation later in infection (data not published). How σ3 functions together with σNS and μNS remains to be investigated. Previous work has shown that the overexpression of σ3 enhances translation of co-expressed mRNAs. The mechanism of enhancement is unclear but is hypothesized to be due to the capacity of σ3 to inhibit PKR [67].

## 5. Compartmentalization of Translation

Viral factories form in the cytoplasm of reovirus-infected host cells that can be seen by immunofluorescence microscopy as early as 4 h postinfection [68,69,70,71,72,73,74,75,76,77,78]. Many RNA viruses induce changes in cellular membranes or assemble inclusion-like structures that serve as the sites of viral replication and assembly. VFs concentrate viral proteins and viral RNAs and perhaps act to prevent their detection by innate immune sensors [79,80,81,82,83]. VFs may sequester components of the innate immune response to prevent downstream signaling. Interferon regulatory factor 3 (IRF3) is sequestered within VFs as a consequence of interactions with μNS preventing it from entering the nucleus and thus impairing the downstream signaling events [84]. Reovirus VFs are the sites of viral replication, transcription, and viral assembly [73,85,86,87]. After virus entry, transcribing core particles become embedded within VFs and viral mRNAs accumulate within them. It was thought that viral mRNAs were translocated out of the VFs into cytoplasm where they engaged ribosomes to allow for the translation and synthesis of new viral proteins, which were then somehow trafficked back into the VFs for viral assembly [73,87]. However, more recent work has shown that reovirus translation likely occurs within or on the margins of VFs [88,89,90]. 

Reovirus VFs are dynamic structures that show liquid–liquid phase-separated properties [88,91]. At 2–4 h postinfection, VFs appear as small, punctate, globular structures which gradually agglomerate and get larger, moving toward the perinuclear region as the infection proceeds [68,91]. When the viral nonstructural protein μNS is expressed ectopically, similar cytosolic globular VF-like structures (VFLs) form, suggesting that μNS nucleates VF formation [77]. μNS acts as a hub for multiple viral protein–protein interactions that are thought to promote viral replication and assembly [75,77,87]. The m3 mRNA, which encodes µNS, is one of four transcripts (s3, s4, m3, and l1) that are expressed very early in infection at high levels before other transcripts are detectable [22]. Indeed, the amount of m3 mRNA expressed is 5 times greater than that of the other three early transcripts (l1, s3, and s4) [22]. Reovirus VFs appear either filamentous or globular depending on the viral strain. A small number of strains form globular VFs [92]. Strains that form globular VFs have a serine at amino acid position 208 in the viral microtubule-associated protein, μ2, whereas strains that form filamentous VFs have a proline at position 208 [74,77]. The viral nonstructural protein σNS is critical for viral replication, and only small punctate VFs form when σNS is knocked down using siRNA [93]. σNS is a single-stranded RNA (ssRNA)-binding protein that localizes to VFs via interactions with the N-terminal 13 amino acids of µNS [75]. When σNS is expressed ectopically, it is diffused in the cytoplasm, but in infected cells, it localizes to VFs concentrating on their periphery [75]. The RNA-binding domain in the N-terminus of σNS is partially required for its interaction with µNS and localization to VFs, suggesting that RNA contributes to the interaction between μNS and σNS. σNS binds RNA nonspecifically and does not appear to bind reovirus RNA preferentially in vitro [24,30]. When bound to RNA, NS forms long, filamentous structures coating the RNA. σNS binding protects both viral RNA and nonviral RNA from degradation [24]. Since σNS localizes to VFs, it is reasonable to hypothesize that σNS sequesters newly transcribed viral mRNAs within VFs and protects them from degradation by the cytoplasmic mRNA decay machinery. It will be interesting to test whether σNS also prevents viral RNA from detection by host defense sensors.

Early thin-section electron microscopy studies reported that ribosomes were absent from reovirus VFs leading to the belief that the translation of reovirus mRNAs occurred outside of VFs [73,87]. However, we found that translational factors and ribosomal components localize to VFs, and ribosomes and membrane fragments could be seen embedded within VFs [88]. Using a ribopuromycylation assay (RPM) to visualize the subcellular localization of actively translating ribosomes, we found incorporation of puromycin into ribosome-associated nascent polypeptides within VFs, suggesting that active translation occurred within VFs [88]. However, a recent study has shown that puromycylated peptides are released and diffused away from the sites of active translation even in the presence of an elongation inhibitor, calling into question our original conclusion that translation occurs within VFs [94]. However, the localization of ribosomal subunits (ribosomal P, pS6R, L11, and rpS3) and translational factors involved in initiation, elongation, termination, and recycling localized to VFs supports the concept that translation may actively occur within VFs. Interestingly, we noted that components of the 43S preinitiation complex (PIC) (eIF3A and pS6R) localize primarily to the outer margins of VFs where they colocalize with σNS. These observations raise the possibility that the 43S PIC are recruited to the margins of VFs and that the initiation of translation occurs there. σNS co-sediments with 40S and 60S ribosomes suggesting an active role of σNS in recruiting ribosomal and translational factors to VFs [95]. The current evidence indicates that translation factors and ribosomes compartmentalize within VFs and that the translation of viral mRNAs likely occurs within or in close association with the margins of VFs. 

Membranes are embedded within reovirus VFs. Studies using transmission electron microscopy, three-dimensional image reconstructions and immunofluorescence confocal microscopy detect ribosome-encrusted and other cellular-membrane fragments within VFs [88,89,90]. The membranous fragments within VFs derive from the endoplasmic reticulum (ER) and are thin, undulated, and fragmented [89,90]. Electron tomography of Tokuyasu cryosections of reovirus-infected cells show that viral particles within VFs appear to be attached to these ER membrane fragments [90]. Based on these observations, Tenorio et al. have suggested that the ER membranes within VFs function to physically support viral replication and viral assembly. However, this hypothesis remains untested. Ectopic co-expression of μNS and σNS recapitulated the ER tubulation and vesiculation seen within infected cells [90]. The findings that rough ER membranes surround and are embedded within VFs support the concept that the translation of viral mRNAs occurs in close association with VFs. 

Ribosomes within cells can be free in the cytoplasm or associated with the ER. During viral infection, mRNA dissociates from free polyribosomes and cellular translation by ER-associated ribosomes are suppressed. However, ribosome loading on ER-bound mRNA is substantially higher than in the cytosol [96], and ER-associated ribosomes can maintain activity under stress that leads to inhibition of translation in the cytosol [97,98,99]. Cap-dependent and -independent translation following Coxsackie B virus infection continues on ER-associated polysomes, indicating that ER-associated ribosomes are relatively protected from the effects of inhibition of cap-dependent translation [99]. Although it was widely believed that ER-bound ribosomes function primarily to synthesize secretory and membraned-bound proteins [100,101], it is now known that ER-bound ribosomes also translate cytosolic proteins, suggesting a role of the ER in global protein synthesis [102]. ER-associated ribosomes recruited to VFs may be used for the translation of viral mRNAs and may be partially protected from the inhibitory effects of phosphorylation of eIF2α. Markers for ER membranes colocalize with σNS at the margins of VFs, together with ribosomal markers, pS6R and ribosomal P [88]. Taken together, these findings support a hypothesis that the translation of reovirus mRNAs preferentially occurs on ER-associated ribosomes. Additionally, a substantial fraction of eIF2α is bound to the ER via a ribosome-independent mechanism [103]. This suggests that the ER may regulate initiation of translation locally. Since ER membranes are associated with VFs, it is feasible that ER-associated translation of viral mRNAs within VFs is able to overcome the inhibition of translational initiation induced by eIF2α phosphorylation. Future work will be required to validate these hypotheses.

By having viral translation occur within or in close proximity to VFs, viral transcription and translation can be coupled, and the requirement for viral mRNA and viral protein trafficking within cells is eliminated. The viral single-stranded RNA-binding protein σNS is concentrated at the margins of VFs and likely plays a role in retaining viral mRNAs within VFs [24,75]. Retention of viral RNA within VFs and association with σNS may protect the viral RNA from degradation and detection by host innate defense sensors [24]. The reovirus dsRNA-binding protein σ3 prevents translational inhibition by inhibiting the activation of PKR. The dsRNA-binding activity of σ3 within VFs might shelter translation from the effects of PKR-mediated phosphorylation of eIF2α. eIF2B, the GTP exchange factor that is inhibited by phosphorylation of eIF2α, also localizes to VFs [88]. VFs may have higher concentrations of ternary complex that the surrounding cytosol, thus allowing translation to proceed despite translational shutdown.

## 6. Role of PKR and RNase L in Translation Suppression

Viral double-stranded RNA (dsRNA) is a pathogen-associated molecular pattern (PAMP) that is recognized by host pattern-recognition receptors (PRRs), which activate downstream signaling pathways leading to interferon (IFN) production and induction of antiviral effectors [104]. By compartmentalizing reovirus transcription, translation and replication within VFs, it is possible that reovirus dsRNA is largely sequestered from host PRRs. Although there is no evidence showing reovirus dsRNA outside of VFs, the dsRNA in rotavirus-infected cells was detected in the cytoplasm outside of viroplasms, the site of rotavirus replication and assembly [105]. Multiple studies have shown that reovirus infection induces type I and type III IFN production and the up-regulation of multiple IFN-stimulated genes [106,107,108]. Viral genome dsRNA or dsRNA formed by secondary structures within viral mRNAs can stimulate a cascade of antiviral events. 

The two best characterized dsRNA-triggered antiviral pathways are the dsRNA-dependent protein-kinase (PKR) pathway and 2′–5′-oligoadenylate synthetase (OAS)/RNase L pathway [109]. PKR is activated upon binding dsRNA leading to its dimerization and subsequent autophosphorylation [110]. Once activated, PKR phosphorylates a variety of substrates to execute its antiviral functions [110]. A major PKR substrate is eIF2α. Phosphorylated eIF2α has an increased binding affinity for the guanine nucleotide exchange factor eIF2B and prevents the recycling of GDP for GTP. Because of limiting amounts of eIF2B, the concentration of charged GTP-bound eIF2 decreases, leading to decreased availability of the ternary complex of eIF2.GTP.met tRNAi and inhibition of translation initiation [60]. PKR can also phosphorylate and activate the kinases IκB kinase and NFκB-inducing kinase, leading to phosphorylation of IκBα and IκBβ and the subsequent activation of the NF-κB pathway [111,112]. In addition to activating PKR, dsRNA activates the 2′–5′ oligoadenylate synthetase (OAS)/RNase L pathway. Upon binding dsRNA, oligoadenylate synthetase (OAS) synthesizes 2′–5′ linked oligoadenylates, which in turn bind and activate RNase L. RNase L cleaves both host and viral RNA substrates and inhibits cellular and viral protein synthesis [113]. Moreover, the cellular and viral RNA cleavage products can be recognized by RIG-I and MDA5 to further amplify the IFN-stimulated antiviral signal. Reovirus infection activates both PKR and OAS/RNase L antiviral pathways [114].

Infection with the most reovirus strains induces host translational shutoff; the Type 2 Jones (T2J) strain or strains clone 8 (c8), clone 87 (c87), and clone 93 (c93) causes dramatic host translational shutoff at high multiplicity [114]. In contrast, infection with the type 3 Dearing (T3D) strain causes minimal effects on host translation. The genetic determinant of the strain-dependent difference in host translational shutoff between T2J and T3D shutoff maps to the reovirus S4 gene segment [115]. The S4 gene encodes the reovirus outer-capsid and dsRNA-binding protein σ3. The association of σ3 with host translational shutoff is attributed to its dsRNA-binding capacity and its role in inhibiting PKR- and RNase L-mediated translational shutoff. The T2J strain requires PKR to induce host translational shutoff, as shutoff does not occur following the infection of mouse embryonic fibroblasts lacking PKR. However, other strains show a dependency on both RNase L and PKR for host shutoff [114]. Current evidence supports the hypothesis that dsRNA binding by σ3 acts to prevent activation of PKR. The translation inhibition induced by overexpression of PKR can be reversed by expression of σ3, but not by σ3 mutants that are defective in dsRNA binding [116,117]. Furthermore, in vitro activation of PKR using dsRNA can be prevented by incubating with extracts prepared from reovirus-infected cells, even if they are treated with interferon prior to collection. The PKR inhibitory activity present in reovirus-infected cell lysates can be blocked if the cell lysate is treated with anti-σ3 serum, [118]. Finally, σ3 has been shown to rescue the replication defects of vaccinia virus [119] and adenovirus mutants [120] that lack their PKR inhibitors, E3L or virus-associated I(VAI) RNA, respectively. Reovirus infection induces PKR and eIF2 α phosphorylation to different extents in a cell- and strain-specific manner [62]. Despite the different effects of reovirus strains on host translation shutoff, all strains can translate viral proteins efficiently in the presence of activated PKR and phosphorylated eIF2 α [62]. How reovirus mRNAs are able to overcome the translation inhibition induced by phosphorylated eIF2α remains unclear. One possibility is that σ3 might locally inhibit PKR and RNase L to preserve viral translation within VFs, while permitting cellular translation to be shutoff. Differences in the binding affinity of σ3 for its partner outer-capsid protein μ1 correlate with the degree of host translational shutoff and the subcellular localization of σ3 to VFs, suggesting that localization of σ3 within infected cells is important for its function [121].

Although it is believed that PKR or RNase L are antiviral factors that are antagonized by the reovirus σ3 protein, it remains controversial to what extent PKR or RNase L exert an antiviral effect during infection. Evidence indicating that phosphorylated PKR has an antiviral effect on reovirus replication comes from experiments investigating the molecular basis for the oncolytic effects of reoviruses on transformed fibroblasts. NIH-3T3 cells are resistant to reovirus infection but become susceptible when transformed with activated Sos or Ras [122]. The authors found that in non-transformed NIH-3T3 cells, the translation of oncolytic T3D viral transcripts was inhibited, and this correlated with the phosphorylation of PKR. However, T3D infection did not induce phosphorylation of PKR in NIH-3T3 cells that were transformed with Sos or Ras. The authors suggested that signaling pathways downstream of Sos and Ras either promoted dephosphorylation of PKR or prevented its activation by reovirus transcripts produced early in infection of NIH-3T3 cells [122]. The authors of this study also compared the capacity of the oncolytic T3D reovirus to synthesize viral proteins in WT and PKR-null mouse embryonic fibroblasts (MEF) and showed that at 48 h postinfection, the PKR-null MEFs produced substantially more viral proteins [122]. In contrast, another study investigating reovirus-induced host shutoff using different reovirus strains found that although both PKR and RNase L contribute to the host shutoff by reovirus infection, neither of them appeared to exert an antiviral effect on reovirus growth [114]. Indeed, some strains (T3D in PKR null MEFs and RNase L-null MEFs, and T2J in RNase L-null MEFs) replicated less efficiently in PKR or RNase L-null cells than in their WT counterparts, suggesting that reovirus may benefit from PKR- and/or RNase L-mediated antiviral signaling [114]. It is important to note differences between the two studies that may explain the differing results. Firstly, the oncolytic strain of T3D is genetically different to the T3D strain used by many labs and importantly has nonsynonomous changes within the S4 gene segment that affect replication and induction of the type I interferon response [108,123]. In addition, the study investigating reovirus-induced host shutoff infected cells at a high multiplicity of infection (80 PFU per cell) [114], whereas the study using the oncolytic strain of T3D used a lower multiplicity (10 PFU per cell) that does not induce host shutoff [122].

Both PKR and RNase L participate in the formation of cytoplasmic RNP granules in response to virus infection. Many viruses induce the formation of SGs [43,124]. Upon treatment of a dsRNA analog poly I:C, activated RNase L promotes widespread mRNA decay and reduces bulk translation, while preserving the translation of antiviral mRNA like IFN- β [125]. The degradation of SG-associated mRNA by RNase L limits SG assembly and leads to the formation of small, punctate, SG-like bodies, that are called RNase L-dependent bodies (RLBs) [126]. RLBs are distinct from canonical SGs and have a different protein composition and no requirement for PKR, eIF2 α phosphorylation, or G3BP1 [126]. One study using 2′–5′ oligoadenylates, a more direct activator of RNase L than poly I:C, found that RNase L promotes the formation of antiviral stress granules (avSGs), which differ again from RLBs in their protein composition and the requirement of PKR, eIF2 and G3BP1 [127]. In addition, 2′–5′ oligoadenylate is a unique ligand for RNase L, whereas poly I:C can activate other dsRNA-binding proteins. Moreover, the level of OAS isoforms (OAS3 is the major isoform for RNase L activation) in different cell types may also contribute to the determinant of the type of granules, either RLBs or avSGs [128]. At early times after reovirus infection, the types of granules induced by reovirus remain incompletely characterized. Studies to date have used one or two protein markers to label the granules, and these markers cannot distinguish between SGs, avSGs, or RLBs, or combinations of these ribonucleoprotein granules. Future studies are needed to characterize the different types of RNP granules and their functions within reovirus-infected cells.

## 7. Viral Proteins that Associate with Ribosomes/Polysomes

Reovirus proteins σNS and σ3 associate with ribosomes or polysomes [30,31,88,95,129,130]. σNS forms a complex that co-sediments with 40–60S ribosomes. This complex contains reovirus RNA and can be dissociated to release free σNS by treatment with 0.5 M potassium chloride [95]. As mentioned before, σNS may play a role in recruiting ribosomes for translation of viral mRNAs, as it can be seen to colocalize along the margins of viral factories with eIF3a, eIF3b, components of small ribosomal subunits such as RiboP, pS6R, and rpS3 [63,88]. In addition, antibodies against eIF3A and pS6R, components associated with the 43S ribosome, co-immunoprecipitate σNS in cells ectopically expressing σNS [88]. Recently, we have found that σNS cofractionates with 40S ribosomes, and, to a lesser extent, with 60S and 80S monosomes and polysomes in infected cells as well as in cells that are ectopically expressing σNS alone (unpublished findings). Even with RNase A treatment, which causes dissolution of polysomes, σNS still cofractionates with 40S ribosomes, suggesting that σNS directly interacts with small ribosomes.

The association of σ3 with ribosomes/ polysomes and its functional significance remains unclear. Late in the infection, some reports have described the synthesis of reovirus mRNAs that are uncapped yet are efficiently translated. Entering core particles synthesize capped viral mRNAs, but newly assembled “secondary” core particles purified from infected cells transcribe noncapped viral mRNAs [131]. It is hypothesized that the capping enzymes within the λ2 protein turret of newly assembled secondary cores are somehow masked. Extracts prepared from reovirus infected cells but not from uninfected cells promote the translation of reovirus uncapped mRNAs. The key factor present in the infected extracts was σ3, as preincubation with an anti-σ3 serum abolished the stimulatory effect present in the infected cell extracts, and purified σ3 was also shown to stimulate the translation of late uncapped viral mRNAs [130]. Notably, the purified σ3 protein did not stimulate or inhibit the translation of reovirus capped mRNAs or cellular mRNAs. σ3 was enriched in the crude initiation-factor fraction that was prepared by a high-salt wash of ribosomes from late-infected cells, or by immunoprecipitation from early-infected cells [129]. The binding of σ3 to ribosomes may be essential for translation of viral late uncapped mRNA. However, other authors did not detect uncapped reovirus mRNAs in infected cells and suggested the phenomenon may be cell-type specific [132]. The association of σ3 with ribosomes/polysomes and its functional significance remain unknown. 

It has been proposed that σ3 may act as a translation-initiation factor that binds to ribosomes to stimulate late uncapped viral translation. When expressed ectopically, σ3 predominantly localizes to the nucleus [133]. Similarly, early after reovirus infection, σ3 can be seen to partially localize to the nucleus before it relocates to VFs. A previous study found that localization of σ3 to the nucleus required its dsRNA-binding activity [133]. The nucleus, specifically the nucleolus, is the main site of ribosome biogenesis [134,135]. It would be interesting to see if σ3 directly binds to ribosomal proteins or other proteins involved in ribosome biogenesis. Although it was suggested that σ3 binds dsRNA nonspecifically [95,117,136], the types of dsRNA or dsRNA regions (for example, the ribosomal RNA) that σ3 binds during reovirus infection have not been studied. It would be worthwhile to thoroughly analyze the dsRNA sequences that are bound by σ3 to better understand possible functions of σ3 during reovirus translation. 

## 8. Concluding Remarks

**Figure 1:**
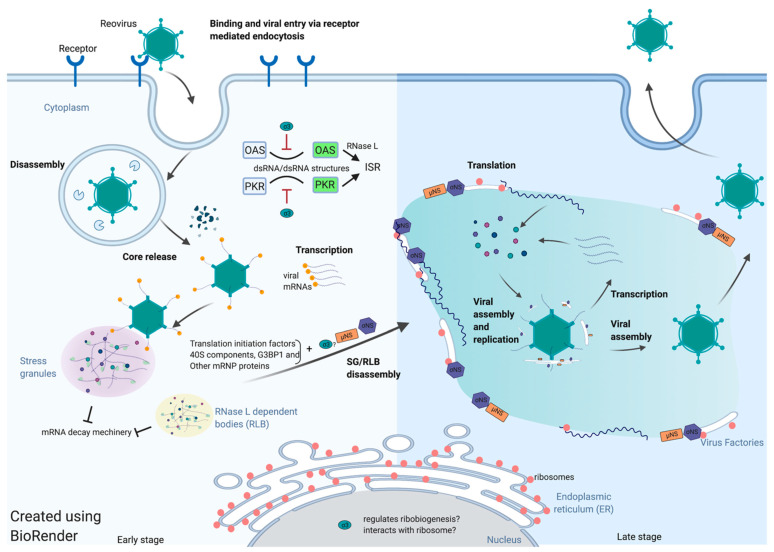
Spatiotemporal regulation of reovirus translation.

Reovirus protein synthesis takes place in the cytoplasm and depends exclusively on the host translational machinery. Therefore, reovirus has developed strategies to promote efficient viral translation in a competitive cellular environment. It seems likely that translation of reovirus mRNAs is spatiotemporally regulated (Figure 1). Evidence in support of this hypothesis includes the observation that early after infections, reovirus induces stress granules and the incoming viral core particles localize to SGs. Localization of entering core particles to SGs requires transcription of viral mRNA [25]. The SGs potentially sequester and protect viral mRNA from the cellular RNA-decay machinery at the very earliest stages of infection. As reovirus infection proceeds, reovirus viral proteins σNS, µNS, and perhaps σ3 seem to be involved in the disruption of SGs even in the presence of eIF2α phosphorylation. A reasonable model is that reovirus usurps SGs to provide access to the translational machinery. The highly concentrated translation-initiation factors, 40S ribosome components and certain mRNP regulators may be recruited to the VFs that are formed gradually during infection. This relocation is mediated by the interaction between σNS and SG protein G3BP1. As VFs form, active translation begins to occur on the margins or within VFs. ER-membrane fragments are found inside the VFs that have been remodeled by the actions of σNS and µNS. The function of the ER within VFs is as yet unknown, but it seems reasonable to surmise that ER membranes act as scaffolds for viral core assembly and replication. Similarly, rough ER membranes and ribosomes at the periphery of VFs may function to support viral mRNA translation.

Our growing understanding of reovirus translation leaves many questions as yet unanswered. Some important questions to be addressed in future studies are as follows:

○ What types of RNP granules or RNP granules combinations are formed at early stages after infection, and what function do RNP granules have during viral infection?
○ Are early viral mRNAs translated within RNP granules?
○ What is the mechanism by which reoviruses disrupt SGs/RNP granules?
○ How does σ3 regulate reovirus translation?
○ What is the functional significance of the nuclear localization of σ3?
○ Are uncapped viral mRNAs synthesized late in infection, and if so, how does σ3 promote their translation?
○ How does the activation of the ISR and PKR or RNase L benefit viral replication?

## Figures and Tables

**Table 1:** Reovirus genome segments, mRNAs, and proteins.

Genome Segment	mRNA	5′-UTR and 3′-UTR Length	Viral Proteins
L1	*l1*	24 nt and 35 nt	λ3 (viral RNA-dependent RNA polymerase)
L2	*l2*	13 nt and 36 nt	λ2 (core structural)
L3	*l3*	13 nt and 63 nt	λ1 (core structural)
M1	*m1*	13 nt and 83 nt	μ2 (polymerase cofactor; microtubule-binding)
M2	*m2*	29 nt and 50 nt	μ1 (outer-capsid structural)
M3	*m3*	18 nt and 60 nt	μNS (nonstructural; viral factory matrix) μNSC (nonstructural)
S1	*s1*	12 nt and 39 nt	σ1 (outer-capsid structural protein; attachment) σ1s (nonstructural)
S2	*s2*	18 nt and 59 nt	σ2 (core structural)
S3	*s3*	27 nt and 73 nt	σNS (nonstructural; single-stranded RNA binding)
S4	*s4*	32 nt and 69 nt	σ3 (outer-capsid structural; dsRNA-binding)

## References

[1] Jackson R.J., Hellen C.U.T., Pestova T.V. (2010). The mechanism of Eukaryotic Translation Initiation and Principles of Its Regulation. Nat. Rev. Mol. Cell Biol..

[2] Schuller A.P., Green R. (2018). Roadblocks and Resolutions in Eukaryotic Translation. Nat. Rev. Mol. Cell Biol..

[3] Sonenberg N., Hinnebusch A.G. (2009). Regulation of Translation Initiation in Eukaryotes: Mechanisms and Biological Targets. Cell.

[4] Hoang H.-D., Neault S., Pelin A., Alain T. (2021). Emerging Translation Strategies during Virus–Host Interaction. Wiley Interdiscip. Rev. RNA.

[5] Chen F., Wu P., Deng S., Zhang H., Hou Y., Hu Z., Zhang J., Chen X., Yang J.-R. (2020). Dissimilation of Synonymous Codon Usage Bias in Virus–Host Coevolution Due to Translational Selection. Nat. Ecol. Evol..

[6] Quax T.E.F., Claassens N.J., Söll D., van der Oost J. (2015). Codon Bias as a Means to Fine-Tune Gene Expression. Mol. Cell.

[7] Mioduser O., Goz E., Tuller T. (2017). Significant Differences in Terms of Codon Usage Bias between Bacteriophage Early and Late Genes: A Comparative Genomics Analysis. BMC Genom..

[8] Fields B.N., Knipe D.M., David M., Howley P.M. (2013). Fields Virology.

[9] Shatkin A.J., LaFiandra A.J. (1972). Transcription by Infectious Subviral Particles of Reovirus. J. Virol..

[10] Watanabe Y., Millward S., Graham A.F. (1968). Regulation of Transcription of the Reovirus Genome. J. Mol. Biol..

[11] Ernst H., Shatkin A.J. (1985). Reovirus Hemagglutinin mRNA Codes for Two Polypeptides in Overlapping Reading Frames. Proc. Natl. Acad. Sci. USA.

[12] Belli B.A., Samuel C.E. (1991). Biosynthesis of Reovirus-Specified Polypeptides: Expression of Reovirus S1-Encoded σ1 NS Protein in Transfected and Infected Cells as Measured with Serotype Specific Polyclonal Antibody. Virology.

[13] Sarkar G., Pelletier J., Bassel-Duby R., Jayasuriya A., Fields B.N., Sonenberg N. (1985). Identification of a New Polypeptide Coded by Reovirus Gene S1. J. Virol..

[14] Busch L.K., Rodríguez-Grille J., Casal J.I., Martínez-Costas J., Benavente J. (2011). Avian and Mammalian Reoviruses Use Different Molecular Mechanisms to Synthesize Their μNS Isoforms. J. Gen. Virol..

[15] Sagar V., Murray K.E. (2014). The Mammalian Orthoreovirus Bicistronic M3 mRNA Initiates Translation Using a 5′ End-Dependent, Scanning Mechanism That Does Not Require Interaction of 5′-3′ Untranslated Regions. Virus Res..

[16] Furuichi Y., Morgan M., Muthukrishnan S., Shatkin A.J. (1975). Reovirus Messenger RNA Contains a Methylated, Blocked 5′-Terminal Structure: M-7G(5’)Ppp(5’)G-MpCp-. Proc. Natl. Acad. Sci. USA.

[17] Luongo C.L., Reinisch K.M., Harrison S.C., Nibert M.L. (2000). Identification of the Guanylyltransferase Region and Active Site in Reovirus mRNA Capping Protein Lambda2. J. Biol. Chem..

[18] Luongo C.L., Contreras C.M., Farsetta D.L., Nibert M.L. (1998). Binding Site for S-Adenosyl-L-Methionine in a Central Region of Mammalian Reovirus Lambda2 Protein. Evidence for Activities in mRNA Cap Methylation. J. Biol. Chem..

[19] Reinisch K.M., Nibert M.L., Harrison S.C. (2000). Structure of the Reovirus Core at 3.6 Å Resolution. Nature.

[20] Narayanan K., Makino S. (2013). Interplay between Viruses and Host mRNA Degradation. Biochim. Biophys. Acta Gene Regul. Mech..

[21] Ng W.C., Soto-Acosta R., Bradrick S.S., Garcia-Blanco M.A., Ooi E.E. (2017). The 5′ and 3′ Untranslated Regions of the Flaviviral Genome. Viruses.

[22] Nonoyama M., Millward S., Graham A.F. (1974). Control of Transcription of the Reovirus Genome. Nucleic Acids Res..

[23] Farsetta D.L., Chandran K., Nibert M.L. (2000). Transcriptional Activities of Reovirus RNA Polymerase in Recoated Cores. Initiation and Elongation Are Regulated by Separate Mechanisms. J. Biol. Chem..

[24] Zamora P.F., Hu L., Knowlton J.J., Lahr R.M., Moreno R.A., Berman A.J., Prasad B.V.V., Dermody T.S. (2018). Reovirus Nonstructural Protein σNS Acts as an RNA-Stability Factor Promoting Viral Genome Replication. J. Virol..

[25] Broering T.J., Kim J., Miller C.L., Piggott C.D.S., Dinoso J.B., Nibert M.L., Parker J.S.L. (2004). Reovirus Nonstructural Protein μNS Recruits Viral Core Surface Proteins and Entering Core Particles to Factory-like Inclusions. J. Virol..

[26] Qin Q., Hastings C., Miller C.L. (2009). Mammalian Orthoreovirus Particles Induce and Are Recruited into Stress Granules at Early Times Postinfection. J. Virol..

[27] Wells S.E., Hillner P.E., Vale R.D., Sachs A.B. (1998). Circularization of mRNA by Eukaryotic Translation Initiation Factors. Mol. Cell.

[28] Groft C.M., Burley S.K. (2002). Recognition of EIF4G by Rotavirus NSP3 Reveals a Basis for mRNA Circularization. Mol. Cell.

[29] Deo R.C., Groft C.M., Rajashankar K.R., Burley S.K. (2002). Recognition of the Rotavirus mRNA 3’ Consensus by an Asymmetric NSP3 Homodimer. Cell.

[30] Gillian A.L., Schmechel S.C., Livny J., Schiff L.A., Nibert M.L. (2000). Reovirus Protein σNS Binds in Multiple Copies to Single-Stranded RNA and Shares Properties with Single-Stranded DNA Binding Proteins. J. Virol..

[31] Gillian A.L., Nibert M.L. (1998). Amino Terminus of Reovirus Nonstructural Protein σNS Is Important for ssRNA Binding and Nucleoprotein Complex Formation. Virology.

[32] Kozak M., Shatkin A.J. (1976). Characterization of Ribosome-Protected Fragments from Reovirus Messenger RNA. J. Biol. Chem..

[33] Kozak M. (1980). Binding of Wheat Germ Ribosomes to Fragmented Viral mRNA. J. Virol..

[34] Kozak M. (1980). Binding of Wheat Germ Ribosomes to Bisulfite-Modified Reovirus Messenger RNA: Evidence for a Scanning Mechanism. J. Mol. Biol..

[35] Kozak M. (1982). Analysis of Ribosome Binding Sites from the S1 Message of Reovirus. Initiation at the First and Second AUG Codons. J. Mol. Biol..

[36] Shatkin A.J. (1974). Methylated Messenger RNA Synthesis In Vitro by Purified Reovirus. Proc. Natl. Acad. Sci. USA.

[37] Furuichi Y., Muthukrishnan S., Shatkin A.J. (1975). 5’-Terminal m-7G(5’)Ppp(5’)G-m-p in Vivo: Identification in Reovirus Genome RNA. Proc. Natl. Acad. Sci. USA.

[38] Ingolia N.T. (2014). Ribosome Profiling: New Views of Translation, from Single Codons to Genome Scale. Nat. Rev. Genet..

[39] Castelló A., Fischer B., Eichelbaum K., Horos R., Beckmann B.M., Strein C., Davey N.E., Humphreys D.T., Preiss T., Steinmetz L.M. (2012). Insights into RNA Biology from an Atlas of Mammalian mRNA-Binding Proteins. Cell.

[40] Shi Z., Barna M. (2015). Translating the Genome in Time and Space: Specialized Ribosomes, RNA Regulons, and RNA-Binding Proteins. Annu. Rev. Cell Dev. Biol..

[41] Bischoff J.R., Samuel C.E. (1989). Mechanism of Interferon Action. Activation of the Human P1/EIF-2 Alpha Protein Kinase by Individual Reovirus s-Class mRNAs: S1 mRNA Is a Potent Activator Relative to S4 mRNA. Virology.

[42] Ivanov P., Kedersha N., Anderson P. (2019). Stress Granules and Processing Bodies in Translational Control. Cold Spring Harb. Perspect. Biol..

[43] Tsai W.-C., Lloyd R.E. (2014). Cytoplasmic RNA Granules and Viral Infection. Annu. Rev. Virol..

[44] Treeck B.V., Protter D.S.W., Matheny T., Khong A., Link C.D., Parker R. (2018). RNA Self-Assembly Contributes to Stress Granule Formation and Defining the Stress Granule Transcriptome. Proc. Natl. Acad. Sci. USA.

[45] Van Treeck B., Parker R. (2018). Emerging Roles for Intermolecular RNA-RNA Interactions in RNP Assemblies. Cell.

[46] Protter D.S.W., Parker R. (2016). Principles and Properties of Stress Granules. Trends Cell Biol..

[47] Yang P., Mathieu C., Kolaitis R.-M., Zhang P., Messing J., Yurtsever U., Yang Z., Wu J., Li Y., Pan Q. (2020). G3BP1 Is a Tunable Switch That Triggers Phase Separation to Assemble Stress Granules. Cell.

[48] Guillén-Boixet J., Kopach A., Holehouse A.S., Wittmann S., Jahnel M., Schlüßler R., Kim K., Trussina I.R.E.A., Wang J., Mateju D. (2020). RNA-Induced Conformational Switching and Clustering of G3BP Drive Stress Granule Assembly by Condensation. Cell.

[49] Kedersha N., Panas M.D., Achorn C.A., Lyons S., Tisdale S., Hickman T., Thomas M., Lieberman J., McInerney G.M., Ivanov P. (2016). G3BP–Caprin1–USP10 Complexes Mediate Stress Granule Condensation and Associate with 40S Subunits. J. Cell Biol..

[50] Kedersha N., Anderson P. (2002). Stress Granules: Sites of mRNA Triage That Regulate mRNA Stability and Translatability. Biochem. Soc. Trans..

[51] Edupuganti R.R., Geiger S., Lindeboom R.G.H., Shi H., Hsu P.J., Lu Z., Wang S.-Y., Baltissen M.P.A., Jansen P.W.T.C., Rossa M. (2017). N 6 -Methyladenosine (m 6 A) Recruits and Repels Proteins to Regulate mRNA Homeostasis. Nat. Struct. Mol. Biol..

[52] White J.P., Lloyd R.E. (2012). Regulation of Stress Granules in Virus Systems. Trends Microbiol..

[53] Hosmillo M., Lu J., McAllaster M.R., Eaglesham J.B., Wang X., Emmott E., Domingues P., Chaudhry Y., Fitzmaurice T.J., Tung M.K. (2019). Noroviruses Subvert the Core Stress Granule Component G3BP1 to Promote Viral VPg-Dependent Translation. eLife.

[54] Lutz M.M., Worth M.P., Hinchman M.M., Parker J.S.L., Ledgerwood E.D. (2019). Mammalian Orthoreovirus Infection Is Enhanced in Cells Pre-Treated with Sodium Arsenite. Viruses.

[55] Kim D.Y., Reynaud J.M., Rasalouskaya A., Akhrymuk I., Mobley J.A., Frolov I., Frolova E.I. (2016). New World and Old World Alphaviruses Have Evolved to Exploit Different Components of Stress Granules, FXR and G3BP Proteins, for Assembly of Viral Replication Complexes. PLoS Pathog..

[56] Chernov K.G., Barbet A., Hamon L., Ovchinnikov L.P., Curmi P.A., Pastré D. (2009). Role of Microtubules in Stress Granule Assembly: Microtubule Dynamical Instability Favors the Formation of Micrometric Stress Granules in Cells. J. Biol. Chem..

[57] McEwen E., Kedersha N., Song B., Scheuner D., Gilks N., Han A., Chen J.-J., Anderson P., Kaufman R.J. (2005). Heme-Regulated Inhibitor Kinase-Mediated Phosphorylation of Eukaryotic Translation Initiation Factor 2 Inhibits Translation, Induces Stress Granule Formation, and Mediates Survival upon Arsenite Exposure. J. Biol. Chem..

[58] Mateju D., Eichenberger B., Voigt F., Eglinger J., Roth G., Chao J.A. (2020). Single-Molecule Imaging Reveals Translation of mRNAs Localized to Stress Granules. Cell.

[59] Carroll K., Hastings C., Miller C.L. (2014). Amino Acids 78 and 79 of Mammalian Orthoreovirus Protein μNS Are Necessary for Stress Granule Localization, Core Protein λ2 Interaction, and de Novo Virus Replication. Virology.

[60] Costa-Mattioli M., Walter P. (2020). The Integrated Stress Response: From Mechanism to Disease. Science.

[61] Smith J.A., Schmechel S.C., Raghavan A., Abelson M., Reilly C., Katze M.G., Kaufman R.J., Bohjanen P.R., Schiff L.A. (2006). Reovirus Induces and Benefits from an Integrated Cellular Stress Response. J. Virol..

[62] Qin Q., Carroll K., Hastings C., Miller C.L. (2011). Mammalian Orthoreovirus Escape from Host Translational Shutoff Correlates with Stress Granule Disruption and Is Independent of eIF2α Phosphorylation and PKR. J. Virol..

[63] Choudhury P., Bussiere L.D., Miller C.L. (2017). Mammalian Orthoreovirus Factories Modulate Stress Granule Protein Localization by Interaction with G3BP1. J. Virol..

[64] Fros J.J., Domeradzka N.E., Baggen J., Geertsema C., Flipse J., Vlak J.M., Pijlman G.P. (2012). Chikungunya Virus nsP3 Blocks Stress Granule Assembly by Recruitment of G3BP into Cytoplasmic Foci. J. Virol..

[65] Scholte F.E.M., Tas A., Albulescu I.C., Žusinaite E., Merits A., Snijder E.J., van Hemert M.J. (2015). Stress Granule Components G3BP1 and G3BP2 Play a Proviral Role Early in Chikungunya Virus Replication. J. Virol..

[66] Götte B., Panas M.D., Hellström K., Liu L., Samreen B., Larsson O., Ahola T., McInerney G.M. (2019). Separate Domains of G3BP Promote Efficient Clustering of Alphavirus Replication Complexes and Recruitment of the Translation Initiation Machinery. PLOS Pathog..

[67] Giantini M., Shatkin A.J. (1989). Stimulation of Chloramphenicol Acetyltransferase mRNA Translation by Reovirus Capsid Polypeptide σ3 in Cotransfected COS Cells. J. Virol..

[68] Becker M.M., Peters T.R., Dermody T.S. (2003). Reovirus σNS and μNS Proteins Form Cytoplasmic Inclusion Structures in the Absence of Viral Infection. J. Virol..

[69] Dales S., Omatos P.J., Hsu K.C. (1965). The Uptake and Development of Reovirus in Strain L Cells Followed with Labeled Viral Ribonucleic Acid and Ferritin-Antibody Conjugates. Virology.

[70] Silverstein S.C., Schur P.H. (1970). Immunofluorescent Localization of Double-Stranded RNA in Reovirus-Infected Cells. Virology.

[71] Silverstein S.C., Dales S. (1968). The Penetration of Reovirus RNA and Initiation of Its Genetic Function In L-strain Fibroblasts. J. Cell Biol..

[72] Sharpe A.H., Chen L.B., Fields B.N. (1982). The Interaction of Mammalian Reoviruses with the Cytoskeleton of Monkey Kidney CV-1 Cells. Virology.

[73] Rhim J.S., Jordan L.E., Mayor H.D. (1962). Cytochemical, Fluorescent-Antibody and Electron Microscopic Studies on the Growth of Reovirus (ECHO 10) in Tissue Culture. Virology.

[74] Parker J.S.L., Broering T.J., Kim J., Higgins D.E., Nibert M.L. (2002). Reovirus Core Protein μ2 Determines the Filamentous Morphology of Viral Inclusion Bodies by Interacting with and Stabilizing Microtubules. J. Virol..

[75] Miller C.L., Broering T.J., Parker J.S.L., Arnold M.M., Nibert M.L. (2003). Reovirus σNS Protein Localizes to Inclusions through an Association Requiring the μNS Amino Terminus. J. Virol..

[76] Mbisa J.L., Becker M.M., Zou S., Dermody T.S., Brown E.G. (2000). Reovirus μ2 Protein Determines Strain-Specific Differences in the Rate of Viral Inclusion Formation in L929 Cells. Virology.

[77] Broering T.J., Parker J.S.L., Joyce P.L., Kim J., Nibert M.L. (2002). Mammalian Reovirus Nonstructural Protein μNS Forms Large Inclusions and Colocalizes with Reovirus Microtubule-Associated Protein μ2 in Transfected Cells. J. Virol..

[78] Becker M.M., Goral M.I., Hazelton P.R., Baer G.S., Rodgers S.E., Brown E.G., Coombs K.M., Dermody T.S. (2001). Reovirus σNS Protein Is Required for Nucleation of Viral Assembly Complexes and Formation of Viral Inclusions. J. Virol..

[79] de Castro I.F., Volonté L., Risco C. (2013). Virus Factories: Biogenesis and Structural Design. Cell. Microbiol..

[80] Netherton C.L., Wileman T. (2011). Virus Factories, Double Membrane Vesicles and Viroplasm Generated in Animal Cells. Curr. Opin. Virol..

[81] Novoa R.R., Calderita G., Arranz R., Fontana J., Granzow H., Risco C. (2005). Virus Factories: Associations of Cell Organelles for Viral Replication and Morphogenesis. Biol. Cell.

[82] Den Boon J.A., Ahlquist P. (2010). Organelle-Like Membrane Compartmentalization of Positive-Strand RNA virus Replication Factories. Annu. Rev. Microbiol..

[83] Nagy P.D., Pogany J. (2012). The Dependence of Viral RNA Replication on Co-Opted Host Factors. Nat. Rev. Microbiol..

[84] Stanifer M.L., Kischnick C., Rippert A., Albrecht D., Boulant S. (2017). Reovirus Inhibits Interferon Production by Sequestering IRF3 into Viral Factories. Sci. Rep..

[85] Lemay G. (2018). Synthesis and Translation of Viral mRNA in Reovirus-Infected Cells: Progress and Remaining Questions. Viruses.

[86] Tenorio R., Fernández de Castro I., Knowlton J.J., Zamora P.F., Sutherland D.M., Risco C., Dermody T.S. (2019). Function, Architecture, and Biogenesis of Reovirus Replication Neoorganelles. Viruses.

[87] Miller C.L., Arnold M.M., Broering T.J., Hastings C.E., Nibert M.L. (2010). Localization of Mammalian Orthoreovirus Proteins to Cytoplasmic Factory-Like Structures via Nonoverlapping Regions of μNS. J. Virol..

[88] Desmet E.A., Anguish L.J., Parker J.S.L. (2014). Virus-Mediated Compartmentalization of the Host Translational Machinery. MBio.

[89] De Castro I.F., Zamora P.F., Ooms L., Fernández J.J., Lai C.M.-H., Mainou B.A., Dermody T.S., Risco C. (2014). Reovirus Forms Neo-Organelles for Progeny Particle Assembly within Reorganized Cell Membranes. MBio.

[90] Tenorio R., de Castro I.F., Knowlton J.J., Zamora P.F., Lee C.H., Mainou B.A., Dermody T.S., Risco C. (2018). Reovirus σNS and μNS Proteins Remodel the Endoplasmic Reticulum to Build Replication Neo-Organelles. MBio.

[91] Bussiere L.D., Choudhury P., Bellaire B., Miller C.L. (2017). Characterization of a Replicating Mammalian Orthoreovirus with Tetracysteine-Tagged μNS for Live-Cell Visualization of Viral Factories. J. Virol..

[92] Yin P., Keirstead N.D., Broering T.J., Arnold M.M., Parker J.S.L., Nibert M.L., Coombs K.M. (2004). Comparisons of the M1 Genome Segments and Encoded μ2 Proteins of Different Reovirus Isolates. J. Virol..

[93] Kobayashi T., Chappell J.D., Danthi P., Dermody T.S. (2006). Gene-Specific Inhibition of Reovirus Replication by RNA Interference. J. Virol..

[94] Enam S.U., Zinshteyn B., Goldman D.H., Cassani M., Livingston N.M., Seydoux G., Green R. (2020). Puromycin Reactivity Does Not Accurately Localize Translation at the Subcellular Level. eLife.

[95] Huismans H., Joklik W.K. (1976). Reovirus-Coded Polypeptides in Infected Cells: Isolation of Two Native Monomeric Polypeptides with Affinity for Single-Stranded and Double-Stranded RNA, Respectively. Virology.

[96] Reid D.W., Nicchitta C.V. (2012). Primary Role for Endoplasmic Reticulum-Bound Ribosomes in Cellular Translation Identified by Ribosome Profiling. J. Biol. Chem..

[97] Stephens S.B., Dodd R.D., Brewer J.W., Lager P.J., Keene J.D., Nicchitta C.V. (2005). Stable Ribosome Binding to the Endoplasmic Reticulum Enables Compartment-Specific Regulation of mRNA Translation. Mol. Biol. Cell.

[98] Unworth H., Raguz S., Edwards H.J., Higgins C.F., Yagüe E. (2010). mRNA Escape from Stress Granule Sequestration Is Dictated by Localization to the Endoplasmic Reticulum. FASEB J..

[99] Lerner R.S., Nicchitta C.V. (2006). mRNA Translation Is Compartmentalized to the Endoplasmic Reticulum Following Physiological Inhibition of Cap-Dependent Translation. RNA.

[100] Palade G. (1975). Intracellular Aspects of the Process of Protein Synthesis. Science.

[101] Palade G.E. (1955). A Small Particulate Component of the Cytoplasm. J. Cell Biol..

[102] Reid D.W., Nicchitta C.V. (2015). Diversity and Selectivity in mRNA Translation on the Endoplasmic Reticulum. Nat. Rev. Mol. Cell Biol..

[103] Jagannathan S., Reid D.W., Cox A.H., Nicchitta C.V. (2014). De Novo Translation Initiation on Membrane-Bound Ribosomes as a Mechanism for Localization of Cytosolic Protein mRNAs to the Endoplasmic Reticulum. RNA.

[104] Ivashkiv L.B., Donlin L.T. (2014). Regulation of Type I Interferon Responses. Nat. Rev. Immunol..

[105] Rojas M., Arias C.F., López S. (2010). Protein Kinase R Is Responsible for the Phosphorylation of eIF2α in Rotavirus Infection. J. Virol..

[106] Sherry B. (2009). Rotavirus and Reovirus Modulation of the Interferon Response. J. Interferon Cytokine Res..

[107] Lostalé-Seijo I., Martínez-Costas J., Benavente J. (2016). Interferon Induction by Avian Reovirus. Virology.

[108] Mohamed A., Konda P., Eaton H.E., Gujar S., Smiley J.R., Shmulevitz M. (2020). Closely Related Reovirus Lab Strains Induce Opposite Expression of RIG-I/IFN-Dependent versus -Independent Host Genes, via Mechanisms of Slow Replication versus Polymorphisms in dsRNA Binding σ3 Respectively. PLoS Pathog..

[109] Sadler A.J., Williams B.R.G. (2008). Interferon-Inducible Antiviral Effectors. Nat. Rev. Immunol..

[110] Williams B.R. (2001). Signal Integration via PKR. Sci. STKE.

[111] Gil J., Rullas J., García M.A., Alcamí J., Esteban M. (2001). The Catalytic Activity of dsRNA-Dependent Protein Kinase, PKR, Is Required for NF-KappaB Activation. Oncogene.

[112] Zamanian-Daryoush M., Mogensen T.H., DiDonato J.A., Williams B.R.G. (2000). NF-ΚB Activation by Double-Stranded-RNA-Activated Protein Kinase (PKR) Is Mediated through NF-ΚB-Inducing Kinase and IκB Kinase. Mol. Cell. Biol..

[113] Silverman R.H. (2007). Viral Encounters with 2′,5′-Oligoadenylate Synthetase and RNase L during the Interferon Antiviral Response. J. Virol..

[114] Smith J.A., Schmechel S.C., Williams B.R.G., Silverman R.H., Schiff L.A. (2005). Involvement of the Interferon-Regulated Antiviral Proteins PKR and RNase L in Reovirus-Induced Shutoff of Cellular Translation. J. Virol..

[115] Sharpe A.H., Fields B.N. (1982). Reovirus Inhibition of Cellular RNA and Protein Synthesis: Role of the S4 Gene. Virology.

[116] Yue Z., Shatkin A.J. (1997). Double-Stranded RNA-Dependent Protein Kinase (PKR) Is Regulated by Reovirus Structural Proteins. Virology.

[117] Denzler K.L., Jacobs B.L. (1994). Site-Directed Mutagenic Analysis of Reovirus σ3 Protein Binding to dsRNA. Virology.

[118] Imani F., Jacobs B.L. (1988). Inhibitory Activity for the Interferon-Induced Protein Kinase Is Associated with the Reovirus Serotype 1 Sigma 3 Protein. Proc. Natl. Acad. Sci. USA.

[119] Beattie E., Denzler K.L., Tartaglia J., Perkus M.E., Paoletti E., Jacobs B.L. (1995). Reversal of the Interferon-Sensitive Phenotype of a Vaccinia Virus Lacking E3L by Expression of the Reovirus S4 Gene. J. Virol..

[120] Lloyd R.M., Shatkin A.J. (1992). Translational Stimulation by Reovirus Polypeptide Sigma 3: Substitution for VAI RNA and Inhibition of Phosphorylation of the Alpha Subunit of Eukaryotic Initiation Factor 2. J. Virol..

[121] Schmechel S., Chute M., Skinner P., Anderson R., Schiff L. (1997). Preferential Translation of Reovirus mRNA by a σ3-Dependent Mechanism. Virology.

[122] Strong J.E., Coffey M.C., Tang D., Sabinin P., Lee P.W.K. (1998). The Molecular Basis of Viral Oncolysis: Usurpation of the Ras Signaling Pathway by Reovirus. EMBO J..

[123] Mohamed A., Clements D.R., Gujar S.A., Lee P.W., Smiley J.R., Shmulevitz M. (2019). Single Amino Acid Differences between Closely Related Reovirus T3D Lab Strains Alter Oncolytic Potency in Vitro and in Vivo. J. Virol..

[124] Miller C.L. (2011). Stress Granules and Virus Replication. Future Virol..

[125] Burke J.M., Moon S.L., Matheny T., Parker R. (2019). RNase L Reprograms Translation by Widespread mRNA Turnover Escaped by Antiviral mRNAs. Mol. Cell.

[126] Burke J.M., Lester E.T., Tauber D., Parker R. (2020). RNase L Promotes the Formation of Unique Ribonucleoprotein Granules Distinct from Stress Granules. J. Biol. Chem..

[127] Manivannan P., Siddiqui M.A., Malathi K. (2020). RNase L Amplifies Interferon Signaling by Inducing PKR-Mediated Antiviral Stress Granules. J. Virol..

[128] Li Y., Banerjee S., Wang Y., Goldstein S.A., Dong B., Gaughan C., Silverman R.H., Weiss S.R. (2016). Activation of RNase L Is Dependent on OAS3 Expression during Infection with Diverse Human Viruses. Proc. Natl. Acad. Sci. USA.

[129] Lemay G., Millward S. (1986). Expression of the Cloned S4 Gene of Reovirus Serotype 3 in Transformed Eucaryotic Cells: Enrichment of the Viral Protein in the Crude Initiation Factor Fraction. Virus Res..

[130] Lemieux R., Lemay G., Millward S. (1987). The Viral Protein Sigma 3 Participates in Translation of Late Viral mRNA in Reovirus-Infected L Cells. J. Virol..

[131] Skup D., Millward S. (1980). mRNA Capping Enzymes Are Masked in Reovirus Progeny Subviral Particles. J. Virol..

[132] Detjen B.M., Walden W.E., Thach R.E. (1982). Translational Specificity in Reovirus-Infected Mouse Fibroblasts. J. Biol. Chem..

[133] Yue Z., Shatkin A.J. (1996). Regulated, Stable Expression and Nuclear Presence of Reovirus Double-Stranded RNA-Binding Protein Sigma3 in HeLa Cells. J. Virol..

[134] Boisvert F.-M., van Koningsbruggen S., Navascués J., Lamond A.I. (2007). The Multifunctional Nucleolus. Nat. Rev. Mol. Cell Biol..

[135] Thomson E., Ferreira-Cerca S., Hurt E. (2013). Eukaryotic Ribosome Biogenesis at a Glance. J. Cell Sci..

[136] Mabrouk T., Danis C., Lemay G. (2011). Two Basic Motifs of Reovirus σ3 Protein Are Involved in Double-Stranded RNA Binding. Biochem. Cell Biol..

